# Co-expression and promoter content analyses assign a role in biotic and abiotic stress responses to plant natriuretic peptides

**DOI:** 10.1186/1471-2229-8-24

**Published:** 2008-02-29

**Authors:** Stuart Meier, René Bastian, Lara Donaldson, Shane Murray, Vladimir Bajic, Chris Gehring

**Affiliations:** 1 Department of Biotechnology, University of the Western Cape, Private Bag X17, Cape Town - Bellville 7535, South Africa; 2 Department of Molecular and Cell Biology, University of Cape Town, Private Bag, Rondebosch 7701, South Africa; 3 South African National Bioinformatics Institute, University of the Western Cape, Private Bag X17, Cape Town - Bellville 7535, South Africa

## Abstract

**Background:**

Plant natriuretic peptides (PNPs) are a class of systemically mobile molecules distantly related to expansins. While several physiological responses to PNPs have been reported, their biological role has remained elusive. Here we use a combination of expression correlation analysis, meta-analysis of gene expression profiles in response to specific stimuli and in selected mutants, and promoter content analysis to infer the biological role of the *Arabidopsis thaliana *PNP, AtPNP-A.

**Results:**

A gene ontology analysis of *AtPNP-A *and the 25 most expression correlated genes revealed a significant over representation of genes annotated as part of the systemic acquired resistance (SAR) pathway. Transcription of these genes is strongly induced in response to salicylic acid (SA) and its functional synthetic analogue benzothiadiazole S-methylester (BTH), a number of biotic and abiotic stresses including many SA-mediated SAR-inducing conditions, as well as in the constitutive SAR expressing mutants *cpr5 *and *mpk4 *which have elevated SA levels. Furthermore, the expression of *AtPNP-A *was determined to be significantly correlated with the SAR annotated transcription factor, *WRKY 70*, and the promoters of *AtPNP-A *and the correlated genes contain an enrichment in the core WRKY binding W-box *cis*-elements. In constitutively expressing *WRKY 70 *lines the expression of *AtPNP-A *and the correlated genes, including the SAR marker genes, *PR-2 *and *PR-5*, were determined to be strongly induced.

**Conclusion:**

The co-expression analyses, both in wild type and mutants, provides compelling evidence that suggests *AtPNP-A *may function as a component of plant defence responses and SAR in particular. The presented evidence also suggests that the expression of *AtPNP-A *is controlled by WRKY transcription factors and WRKY 70 in particular. *AtPNP-A *shares many characteristics with PR proteins in that its transcription is strongly induced in response to pathogen challenges, it contains an N-terminal signalling peptide and is secreted into the extracellular space and along with PR-1, PR-2 and PR-5 proteins it has been isolated from the Arabidopsis apoplast. Based on these findings we suggest that *AtPNP-A *could be classified as a newly identified PR protein.

## Background

Natriuretic Peptide (NP) systems have been identified in mammals, fish, amphibians, birds and reptiles. NPs and their receptors are commonly associated with organs involved in cardiac and osmoregulatory homeostasis. In amphibians, birds and fish, NPs have been shown to play a critical role in the regulation of blood fluid volume and composition [1].

The first indication that NPs function in plants came from radio-immuno assays on plant tissue extracts from Florida beauty [2] and it was shown that the rate of transpiration, solute flow and solute uptake in carnation and chrysanthemum was rapidly and significantly increased after exogenous application of synthetic human atrial NP (ANP) [3]. Subsequently it was demonstrated that rat ANP can induce stomatal opening in a concentration dependent manner [4] and this effect appears to be dependent on the intracellular second messenger cGMP (guanosine 3',5'-cyclic monophosphate) since it is inhibited by the guanylate cyclase inhibitor LY 83583, but can be induced by the synthetic cell permeant cGMP analogue 8-Br-cGMP [5-7]. Binding experiments of ANP to isolated leaf membranes provide evidence for specific receptor ligand interactions [8].

A plant NP (PNP) from *A. thaliana *(AtPNP-A) and several closely related sequences in different species have since been identified [9,10]. AtPNP-A, its most closely related sequence AtPNP-B, and orthologues in other higher plant species, share a family-45 glucosidase domain with the cell wall loosening expansins [11] and are related to expansins on the basis of this structural homology [9,12]. AtPNP-A (At2g18660) is a small protein of 126 amino acids in length (MW: 14016 kD; pI: 9.22) that is encoded by a gene with a single intron of 100 bp. The region most conserved between PNPs from different plant species has also been shown to be the key to its physiological activity [13]. Evidence for systemic mobility of PNPs comes from the structure and processing of the molecules [9]. The protein contains an N-terminal 24 amino acid signal peptide (MW: 2249) that directs the molecule into the extracellular space and PNPs that are recognized by anti-human atrial natriuretic polypeptide rabbit serum have been localised *in situ *in conductive tissue [14] and were isolated from xylem sap [15], and proteomics studies have identified the AtPNP-A protein in the apoplastic space in *A. thaliana *[16].

**Figure 1:**
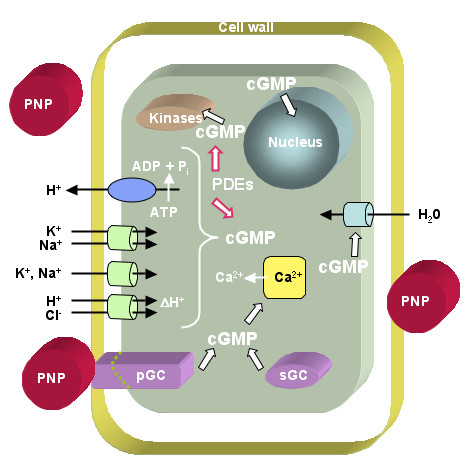
**Model of AtPNP-A action at the cellular level**. The model proposes that AtPNP-A can dock to receptor-like molecules that directly act as particulate guanylyl cyclases (pGCs) or indirectly activate soluble GCs (sGCs). GCs catalyse the reaction from GTP to cGMP. The latter acts as second messenger affecting cytosolic Ca^2+ ^levels, modulating ion channels, activating phosphorylation through kinases and influences the transcriptome. Phosphodiesterases (PDEs) in turn metabolise cGMP to GMP (adapted from [21]).

A number of physiological and biochemical studies have implicated AtPNP-A with a role in the regulation of ion and solute homeostasis. Immuno-reactive PNP (irPNP) extracts and recombinant AtPNP-A have been shown to induce swelling in leaf mesophyll protoplasts [17] and in protoplasts isolated from *Arabidopsis *cell suspension cultures respectively [15]. Further, irPNP rapidly and specifically induced a transient elevation of cGMP levels in the conductive stele tissue of maize roots [6] and in stomatal guard cell protoplasts [7] and recently recombinant AtPNP-A was shown to stimulate protoplast swelling in a cGMP dependent manner [18]. IrPNPs also modulate ion fluxes across plant membranes [19] and recombinant AtPNP-A induced spatially dependent H^+^, K^+ ^and Na^+ ^fluxes in *A. thaliana *roots [20]. Endogenous levels of irPNP are increased in response to NaCl stress in whole-plants and in *Arabidopsis *suspension culture cells exposed to high salt or osmoticum [15]. Collectively, these studies indicate that PNP-like molecules may function as extracellular signalling molecules that directly affect water and solute transport in response to stress. Based on biochemical and physiological data we propose mechanisms of action for AtPNP-A at the cellular level as summarised in Figure 1 (adapted from [21]).

Despite an increasing body of physiological and biochemical data [21], the biological role of this systemically mobile peptide has remained elusive. In order to infer a biological role for AtPNP-A, we made use of the large repositories of *A. thaliana *microarray data to study the expression profiles of *AtPNP-A *and the 25 most expression correlated genes in response to various treatments as well as in mutants. We further analysed the promoters of these genes for known regulatory motifs. The results of our study predict a function for AtPNP-A in plant abiotic and biotic stress responses, and in particular in systemic acquired resistance (SAR). Furthermore, we demonstrate how computational analyses that link regulatory potential as encoded by promoter elements and expression data can provide novel insights into the function of a specific gene as well as groups of genes.

## Results and Discussion

### Expression Correlation and GO Analyses

In the first step of the analyses we extracted and ranked the 25 genes whose expressions are most tightly correlated with *AtPNP-A *(Table 1). The moderate correlation (*r*) values of the listed genes (maximum *r *= 0.73) may reflect that the expression of *AtPNP-A *is subject to complex combinatorial control via multiple promoter motifs with complex inputs from multiple, potentially antagonistic, signalling pathways.

**Table 1:** List of genes that are expression correlated with *AtPNP-A *(At2g18660)

**Locus**	***r*-value**	**Annotation**
At3g57260 ^RBS, SAR^	0.731	Pathogenesis-related protein 2 (PR-2)
At5g10760	0.681	Aspartyl protease family protein
At2g04450 ^RBS^	0.676	Triphosphatase activity, stress response
At5g52760	0.661	Heavy-metal-assoc. domain-containing
At2g17040	0.659	No apical meristem (NAM) family protein
At5g55450 ^RBS^	0.647	Protease inhibitor/lipid transfer protein
At1g21250	0.645	Wall-associated kinase 1 (WAK1)
At4g23610	0.641	Hin1 – role in hypersensitive response
At1g13470	0.634	Mitochondrial protein of unknown function
At4g14365	0.634	Zinc finger (C3HC4-type RING) family
At1g73800	0.630	Calmodulin binding protein
At3g56710	0.629	SigA-binding protein, plastid sigma factor
At4g04490	0.627	PK family, liposaccharide biosynthesis
At2g14560	0.626	Protein of unknown function (DUF 567)
At2g14610 ^RBS, SAR^	0.626	Pathogenesis-related protein 1 (PR-1)
At1g21520	0.626	Expressed protein
At2g24850	0.626	Aminotransferase, resp. to wounding & JA
At4g23150	0.625	Protein kinase family protein
At3g60420	0.622	Phosphohistidine phosphatase activity
At2g32680	0.620	Disease resistance, leucine rich-repeats
At1g74440	0.614	Similar to YGL010w-like protein
At1g02450 ^RBS, SAR^	0.613	NPR1/NIM1-interacting prot. 1 (NIMIN1)
At4g11890	0.606	Protein kinase family protein
At1g75040 ^RBS, SAR^	0.604	Pathogenesis-related protein 5 (PR-5)
At1g08450	0.602	Calreticulin 3 precursor, Ca^2+ ^binding

SAR: Systemic acquired resistance; RBS: Responsive to biotic stress

In order to identify a functional role of *AtPNP-A*, the correlated genes were analysed in FatiGO+ [22-24] to identify any bias in GO functional annotation terms in the correlated list (list 1) compared to the remainder of the *A. thaliana *genome [see Additional file 1]. In the GO search category of biological process there is a significant (Family Wise Error Rate – FWER) adjusted p-value) enrichment in genes involved in biotic defence responses at a number of levels. The most notable bias being at level 8 with a significant (adjusted p-value 0.0000038) enrichment in genes involved in SAR (Table 1). SAR is an inducible plant defense response against local pathogen infection that gives rise to a systemic long lasting resistance to a broad range of virulent pathogens [25]. The SAR response is characterised by the accumulation of endogenous salicylic acid (SA) in infected tissues and later in distal uninfected tissues with a subsequent induction of a select group of pathogenesis-related genes (PR genes) [26].

The enrichment in SAR annotated genes in our list is particularly striking considering that in FatiGO the entire *A. thaliana *genome contains only 21 annotated SAR genes and four of these are present in our list of correlated genes. The four correlated SAR genes include *NIMIN1 *(At1g02450; *r *= 0.61) that is involved in the transcriptional regulation of PR genes [26], *PR-1 *(At2g14610; *r *= 0.63); *PR-2 *(At3g57260; *r *= 0.73) and *PR-5 *(At1g75040; 0.61) whose expression is commonly used as diagnostic markers of the SA dependent SAR response [27]. An extended correlation analysis revealed that an additional 11 SAR annotated genes, including *NPR1 *(or *NIM1*; At1g64280; *r *= 0.52), which is an essential key positive regulator of signal transduction leading to the SAR response and expression of PR proteins, are significantly correlated (9 positive, 2 negative; p < 0.01; bivariate normal distribution) with the expression of *AtPNP-A *[see Additional file 2]. Other correlated genes in list 1 annotated to be involved in plant defence responses and response to biotic stimuli include a disease resistance family protein containing leucine rich-repeats (At2g32680), a stress responsive gene with triphosphatase activity (At2g04450) and a protease inhibitor (At5g55450). The GO analysis for the cellular component and molecular function category revealed no significant difference in biologically relevant labels between the two lists.

The results of the Swiss-Prot keyword search also identified a significant enrichment in genes annotated as PR proteins (adjusted p value = 0.002), involved in signalling (adjusted p value = 0.026) and associated with the apoplast (adjusted p value = 0.040) in list 1. It was noted that along with *PR-1*, *PR-2 *and *PR-5*, *AtPNP-A *is one of six genes in list 1 annotated as having signalling function.

### Microarray Expression Profiles

**Figure 2:**
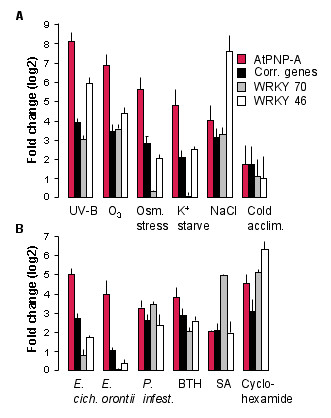
**Expression profile of *AtPNP-A *and correlated genes in response to selected treatments**. The results presented illustrate the fold change (log2) in expression of *AtPNP-A*, *WRKY 70 and WRKY 46 *and the average fold change for the 25 correlated genes in response to abiotic stresses (A) and biotic and chemical treatments (B). (A) The treatments were: UV-B shoot 3 h (n = 2); O_3 _6 h (n = 3); Osmotic stress in the shoot after 3 h (n = 2); K^+ ^starvation in the shoot after 7 days (n = 3); NaCl in the roots after 6 h (n = 2) and cold acclimation after 14 days (n = 3). (B) The treatments were: *Erysiphe cichoracearum *3 days after inoculation (n = 4); *Erysiphe orontii *3 days after inoculation (n = 2); *Phytophthora infestans *1 day after inoculation (n = 3); BTH after 8 h (n = 3); SA after 3 h (n = 2) and cyclohexamide after 3 h (n = 2). Error bars represent standard errors of the mean.

The over representation of genes involved in defence responses, and specifically SAR is consistent with the observation that *AtPNP-A *and the correlated genes are most highly expressed in microarray experiments where defence responses are elicited. The treatments that induce up-regulation of *AtPNP-A *and the correlated genes more than two-fold include SA and other SAR inducing conditions as well as a number of abiotic stresses (Figure 2).

The strong up-regulation of *AtPNP-A *and correlated genes by SA and benzothiadiazole S-methylester (BTH), a synthetic functional SA analogue [28], is a key indicator that these genes are involved in plant defence and specifically SAR since SA has been shown to be essential and sufficient to induce the SAR response in plants [29] (Figure 2B). In addition to *AtPNP-A*, all of the 25 correlated genes were significantly (ANOVA p-value < 0.05) up-regulated by more than two fold after 8 h and 24 h treatments with 60 μM BTH (Supplementary Table 3 in [28]) further linking these genes to the SAR defense pathway. Expression of *AtPNP-A *is also significantly correlated with the isochorismate synthase-1 (*ICS-1*) gene (At1g74710; *r *= 0.50) that is critical for SA biosynthesis [29].

The biotic stresses that induced the largest increase in expression of *AtPNP-A *include infection with the biotrophic pathogens *Phytophthora infestans, Erysiphe cichoracearum *and *Erysiphe orontii *that depend on living host tissue for survival (Figure 2B). Activation of *AtPNP-A *expression and the correlated genes in response to these pathogens is in accord with the literature informing that SA-dependent defenses generally act against biotrophs in contrast to jamonic acid- and ethylene-dependent responses that counteract necrotrophs [30].

The large increases in gene expression induced by ozone and UV-B (+6.86_log2 _and +8.09_log2 _respectively for *AtPNP-A*) is consistent with these genes being part of the SAR response since both these treatments have previously been shown to stimulate SA production and induce the expression of PR genes [31-34].

The expression of *AtPNP-A *and the correlated genes is also strongly modulated by a number of abiotic stresses including K^+ ^starvation, osmotic, and NaCl stress as well as cold acclimation (Figure 2A). A common element of abiotic stresses is that they decrease water potential [35]. It is noteworthy that the induction of *AtPNP-A *in response to ion and osmotic stresses is tissue specific with the response to high Na^+ ^being specific to root tissue with little change observed in shoots while both low K^+ ^and high osmolarity induced elevated transcription in shoots only [36]. This is of interest since the AtPNP-A protein has been shown to affect water movement in shoots [37] and protoplasts [17] as well as ion fluxes in roots [20]. It is thus tempting to speculate that AtPNP-A may have a role in maintaining plant water and ion homeostasis under stress conditions.

K^+ ^is the key inorganic ion required in high quantities by plants while Na^+ ^on the other hand is toxic at high concentrations [38]. Na^+ ^is able to compete with K^+ ^ions for uptake and binding sites thus maintaining the correct Na^+^/K^+ ^ratio in plants is of the utmost importance [39]. Decreases in K^+ ^might cause the plant to take up more Na^+ ^in order to maintain adequate osmotic pressure [40]. Therefore either the increase in cytosolic Na^+ ^or a decrease in osmotic pressure as a consequence of K^+ ^starvation or a combination of both may cause *AtPNP-A *induction.

Elevated expression of *AtPNP-A *and correlated genes, particularly defence genes and SAR annotated genes, by abiotic osmotic stresses as well as defence eliciting treatments may well reflect that both types of challenges lead to common homeostatic disturbances which in turn transcriptionally activate a set of common response genes. This concept is supported by several studies that recognise a role of SA in abiotic stresses such as drought, salinity and temperature [41,42] and the accumulation of PR proteins is in fact a common plant response to both abiotic and biotic stresses further highlighting the overlap in biotic and abiotic defence mechanisms [43].

The generation of reactive oxygen species and changes in ion fluxes have been identified as early responses to both abiotic and biotic stresses, including an influx of H^+ ^and Ca^2+ ^and an efflux of K^+ ^and Cl^- ^[35]. AtPNP-A has been shown to modulate H^+^, Na^+ ^and K^+ ^fluxes [20] thus further implicating AtPNP-A in plant stress responses as do studies which indicate that AtPNP-A signals via the intracellular second messenger cGMP [5,6,18] since cGMP has been shown to be an important signaling molecule in pathogen [44] and osmotic stress responses [45] in plants. It seems particularly relevant that the expression of a gene encoding a cyclic nucleotide-gated channel (CNGC20; At3g17700), which has been shown to be involved in the transport of Ca^2+ ^and K^+^, and in some cases Na^+^, across cell membranes is also correlated with that of *AtPNP-A *(*r *= 0.60) [see Additional file 2] since ion conductance in these channels is regulated by cGMP as well as Ca^2+ ^and calmodulin. These channels have also been implicated in regulating SA-dependent biotic defense responses [46].

*AtPNP-A *expression is also correlated with a number of Ca^2+ ^sensing/binding proteins including, the above mentioned CNGC20, calreticulin 3 (At1g08450; *r *= 0.60), two calmodulin-binding proteins (At1g73800; *r *= 0.63 and At1g73805; *r *= 0.588) with family members involved in the induction of plant defense responses (NCBI sequence viewer, pfam07887) and a Ca^2+^-binding EF hand domain containing protein (At3g47480; *r *= 0.59). One of the earliest responses to biotic and abiotic stresses is an increase in cytosolic free Ca^2+^[47] that in turn plays a role in activating the oxidative burst after elicitor treatment [48,49] and is also linked to signaling SA-induced PR gene expression [50]. The expression of Ca^2+ ^sensing molecules is rapidly induced in response to biotic and abiotic stresses [51] and functions to decode Ca^2+^signatures and/or relay signals to downstream targets, including kinases, which further amplify the Ca^2+ ^signal by inducing downstream phosphorylation cascades [38]. The presence of three kinases (At4g04490; *r *= 0.63, At4g23150; *r *= 0.63 and At4g11890; *r *= 0.61) in amongst the correlated genes (Table 1) is entirely consistent with such a signaling cascade. Moreover, the expression correlation of three stress responsive mitogen-activated protein kinase (MAP kinase) genes *MAPKK *(At4g26070; *r *= 0.59), *MPK 11 *(At1g01560; *r *= 0.58) and *MAPKK *(At4g29810; *r *= 0.57) to *AtPNP-A *also ties in with the proposed cascades [see Additional file 2]. Activation of MAPKs has indeed been reported after exposure to pathogens [52] as well as a number of abiotic stresses [53].

While transcriptional responses to some stresses, including the osmotic, salt, UV-B and some of the biotic treatments, were measured over multiple time points, the data presented here are generally the earliest time point that induced the largest increase in *AtPNP-A *expression. The expression of *AtPNP-A *in some cases showed induction at earlier time points than considered in this study, however, in all cases the expression of *AtPNP-A *generally increased over time and thus high transcript levels were sustained for the duration of the stress, e.g. five days for *E. orontii *and 24 h for osmotic, salt and UV-B treatments (data not shown). The UV-B experiment can be distinguished from the other experiments in that the stress was not maintained for the duration of the experiment. Rather, plants were irradiated for 15 minutes before being transferred back to the standard phytochamber conditions until harvest. The expression of *AtPNP-A *in shoots was elevated at 30 minutes (1.84_log2_; data not shown), peaked at 3 h (8.09_log2_, > 250 fold) (Figure 2A) and remained elevated (6.46_log2; _data not shown) at 24 h after irradiation. This documents that *AtPNP-A *expression remains very high and sustained after the stress has been removed and thus may indicate that the initial damage inflicted, and not the actual presence of the stress itself, is the driving force for the maintained transcriptional activation.

The increase in expression of *AtPNP-A *(4.49_log2_) in response to the protein synthesis inhibitor cycloheximide (CHX) implies that transcription of *AtPNP-A *can occur independently of *de novo *protein synthesis and that concurs with the definition of immediate early response genes [54] that have been proposed to play important roles in the early regulation of defence responses [55]. It has been postulated that CHX induces gene expression via dual mechanisms; by preventing synthesis or activation of a short-lived transcriptional repressor or by removing specific labile transcript degrading enzymes [56]. There is evidence that the induced expression of genes encoding secreted proteins, such as *AtPNP-A *does not require *de novo *protein synthesis [57]. The ability to rapidly induce expression of *AtPNP-A *independently of *de novo *protein synthesis thus implies both an important and early role for this gene in response to specific elicitors.

### Common Motifs in cis and Transcription Factors

The common expression profiles of *APNP-A *and the 25 correlated genes in response to both biotic and abiotic stresses suggests that these genes are under common regulatory control and are thus likely to share common *cis*-elements in their promoter regions. To reveal aspects of common transcriptional activation we analyzed promoter regions of these genes 1 kb upstream of the predicted transcription start site (TSS) for the presence of known plant cis-elements.

**Figure 3:**
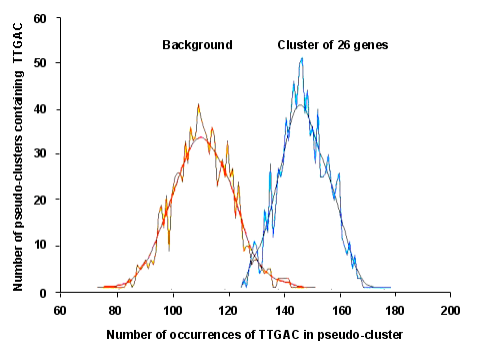
**Frequency of occurrence of the W-box (TTGAC) core motif in artificial clusters generated in POBO for *A. thaliana *background promoters compared to the promoters of AtPNP-A and the 25 most positively expression correlated genes**. The 1 kb upstream promoter sequences of the 26 expression correlated genes were analysed in POBO (see methods and [see Additional file 3]) to determine the frequency of occurrence of the TTGAC W-box core motif. The analysis determined that compared to the *A. thaliana *background (2.24 copies/promoter), there was a significant (t-test: p-value > 0.0001) enrichment in the frequency of the TTGAC motif in our dataset (2.99 copies/promoter).

The analysis in POBO [58] indicated that the invariant core TTGAC W-box motif was present in 25/26 of our correlated genes a total number of 78 times at an average of 2.99 copies/promoter compared to the average of 2.24 across all *A. thaliana *promoters (t-test p-value >0.0001) (Figure 3 and [see Additional file 3]). The analysis in Athena [59] identified that the extended and more stringent TTGAC(A/T) W-box motif was present in 22/26 genes a total of 54 times at an average of 2.08 copies/promoter (p-value = 0.0037; data not shown). Although in Athena this p-value does not qualify the W-box motifs to be enriched in our correlated genes according to the stringent enrichment threshold of <10^-4 ^(Bonferroni correction), it does show that a very high percentage of our genes contain multiple copies of the stringent W-box. Both these promoter analysis methods indicate that multiple copies of the W-box elements are present in a high percentage of our correlated genes with the core TTGAC motifs being significantly enriched compared to expected frequencies in the *A. thaliana *genome suggesting that they are important regulatory elements in these expression correlated genes.

In plants, W-box cis-elements are known to bind WRKY TFs [60] indicating that these TFs may be important in regulating the expression of the correlated genes. This is completely consistent with our expression analysis results since the WRKY family of TFs [60] have well established roles in regulating disease responses in plants [61]. In addition, they have also been documented to mediate abiotic plant responses to freezing [62], wounding [63], oxidative stress [64], drought, salinity, cold, and heat [65-67]. In our study, expression of *AtPNP-A *is moderately correlated with the expression of *WRKY 70 *(At3g56400; *r *= 0.60) and *WRKY 46 *(At2g46400; *r *= 0.56) [see Additional file 2]. When viewing the expression profiles of *WRKY 70 *and *WRKY 46 *genes it is apparent that the various treatments which induced large increases in the expression of the correlated genes in Table 1 also induced marked changes in the expression of the *WRKY *genes (Figure 2). This links the expression of *AtPNP-A *and the *WRKY *genes to common biological responses and raises the possibility that WRKY 70 and WRKY 46 may positively regulate *AtPNP-A *transcription.

While transcription of *WRKY 70 *and *WRKY 46 *is generally strongly induced in response to SAR eliciting treatments, only *WRKY 46 *is consistently co-expressed with *AtPNP-A *in response to the abiotic (ion and osmotic) stresses (Figure 2A and 2B). As previously described for *AtPNP-A *and the correlated genes, the induced expression of *WRKY 46 *is specific to shoots in response to K^+ ^starvation and osmotic stress and to roots in response to NaCl stress.

The expression correlation between *AtPNP-A *and the discussed *WRKY *genes and the overrepresentation of W-boxes in the correlated genes prompted a manual analysis of the promoter of *AtPNP-A *which revealed the presence of four copies of the core TTGAC W-box motif and two occurrences of more stringent TTGAC(C/T) motif clustered in close proximity (starting at -738 and -775) relative to the predicated TSS. The result of the manual inspection coincided with the results returned form Athena and POBO. Similar frequencies of these motifs were observed in a study of 26 SAR regulated genes (termed *PR-1 *regulon genes) in which only W-boxes were present in the promoters of all 26 genes at an average of 4.3 copies of the core and 2.1 copies of the more stringent W-box elements within 1100 bp upstream of the predicated TSS [60]. These values represent a significant enrichment in W-boxes since these authors determined that the statistical expectation for a randomly distributed pentamer (TTGAC) was 2.1 copies and for the hexamer (TTGAC(C/T)) 1.1 copies per 1100 bp of promoter [60].

In summary, the presented evidence is entirely consistent with transcription of *AtPNP-A *and the correlated genes being positively regulated by WRKY TFs. The promoter of *AtPNP-A *and the correlated genes contain an enrichment of the core W-box motif and expression of *AtPNP-A *is correlated with two WRKY genes in response to various SAR eliciting and biotic and abiotic stresses. The correlation of *WRKY 46 *in response to ion and osmotic abiotc stresses was both treatment and tissue specific. In the light of these facts we suggest that the expression of *AtPNP-A *may be closely regulated by WRKY TFs in response to SAR-inducing and abiotic stresses.

### Insights from AtPNP-A Expression in Mutants

The link between AtPNP-A, SA signalling and the WRKY TFs is also supported by expression profiles of *AtPNP-A *and the correlated genes in mutants including a *WRKY 70 *over-expresser and various SAR related mutants present in the mutant surveyor in Genevestigator [68].

**Figure 4:**
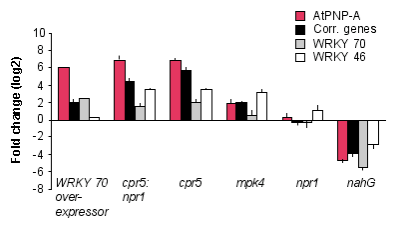
**Expression profile of *AtPNP-A *and selected correlated genes in selected *A. thaliana *mutants**. The expression profiles of *AtPNP-A *and the correlated genes were examined in a number of SA/SAR related mutants. The expression of *AtPNP-A *and selected genes is greatly elevated in *WRKY 70 *over-expresser lines and in mutants with elevated SA levels such as *cpr5 *and *mpk4*. Conversely, in the SA deficient mutant *nahG*, expression of the selected genes is markedly reduced. Error bars represent standard errors of the mean.

WRKY 70 is a SAR annotated TF in FatiGO and has been shown to be an essential factor in plant defense responses necessary for the induction of *PR *gene expression in *A. thaliana *[28,69]. In a microarray study, a constitutive over-expressor of *WRKY 70 *was shown to induce constitutive expression of SA induced PR genes and five of our correlated genes, including PR-2 and PR-5, which are widely considered SAR marker genes, correspond to genes in this study that were up-regulated > 2.5 fold compared to controls [69]. While the 8K Affymetrix chip used for this study did not contain an *AtPNP-A *representative sequence, in an unpublished experiment using the 24K chip, *AtPNP-A *was up-regulated over 50 fold in over-expressing *WRKY 70 *lines and was amongst the top 20 genes that are up-regulated in this study (Figure 4; Personal communication: Gunter Brader, Faculty of Biosciences, University of Helsinki). Additionally, a strong induction in expression of the 25 correlated genes is also observed in this experiment providing further evidence that indicates that WRKY 70 may positively regulate the expression of *AtPNP-A *and the expression correlated genes.

In the *cpr5 *(constitutive expresser of pathogenesis related genes) and *mpk4 *mutants that have elevated levels of SA and display constitutive expression of PR genes [70,71], the expression of *AtPNP-A *and the correlated genes was markedly elevated (Figure 4). It is of particular interest that the four listed mutants that displayed the largest increase in the expression of *AtPNP-A *in Genevestigator are all *cpr5 *mutants, being *cpr5/scv1*, *cpr5/npr1*, *cpr5*, *cpr5/npr1/svi1 *(range +7.55 to +6.21_log2_) (data not shown).

Conversely, the *nahG *mutant that is defective in SA production and signalling, is the only experiment presented in this study that documents a large reduction in the expression of *AtPNP-A *(-4.6_log2_) and the correlated genes (Figure 4). This experiment was performed in senescing leaves [72] to identify SA-dependent global gene expression patterns during developmental senescence since SA has previously been shown to be required for expression of some senescence-induced genes [73]. In this study, and in the ATGE developmental series of *A. thaliana *microarray experiments [74] transcript levels of *AtPNP-A *were elevated approximately 2.8 fold in senescing leaves compared to adult green leaves (data not shown) indicating that *AtPNP-A *is a senescence enhanced gene. Furthermore, since transcript levels of *AtPNP-A *were reduced beyond detection limits in senescing leaves in the *nahG *mutant this induction appears to be SA-dependent. This pattern is completely consistent with other results since premature senescence, including leaf yellowing and necrosis can be induced by biotic and aboitic stresses that stimulate SA production, including ozone [75] and UV-B [76] which also induce large increases in expression of *AtPNP-A*. Thus, there is evidence documenting induction of *AtPNP-A *expression in SA-mediated natural developmental and stress activated processes which both culminate in cell death indicating that *AtPNP-A *may be involved in these processes. The mutant analyses further enforce that the transcriptional regulation of *AtPNP-A *and the correlated genes is largely controlled by SA.

### The role of a TGA TF in PR gene expression

Additional evidence for co-regulation of *AtPNP-A *with SAR annotated genes is provided by the observation that expression of another SAR annotated TF, the *TGA3 *bZIP TF (At1g22070), is correlated with that of *AtPNP-A *(*r *= 0.49). Upon induction of SAR, NIMIN1 and NPR1 form a ternary complex with TGA factors in the nucleus which enhances their binding to the positive regulatory as-1 (activator sequence 1) or as-1-like (TGACG) *cis*-elements that are present in the promoter of several plant genes activated during defense, including *A. thaliana PR-1 *[77-79]. A manual inspection of the *AtPNP-A *promoter identified two occurrences of the TGACG motif in close proximity to the TSS (start at -94 and +24). The correlation in expression between *AtPNP-A*, *NPR1*, *NIMIN1 *and *TGA3 *(Table 1, and [see Additional file 2]) along with the identification of TGA3 *cis*-elements in the promoter of *AtPNP-A *is strong evidence that these two factors contribute to the regulation of *AtPNP-A *expression.

### AtPNP-A as PR Protein

Based on the above results we suggest that AtPNP-A could be classified as a PR protein since it possesses many of the criteria that define this class of proteins. The name "pathogenesis related protein" is a collective term that encompasses all proteins that are present at almost undetectable levels in healthy tissue but are induced at the protein level following pathogen infection. The classification of these proteins is based on their pathogen inducible expression rather than defined functional roles in defence [43]. This point is brought into focus when considering PR-1, which is the quintessential marker of the SAR response yet its biological role is largely unknown [80]. Although *AtPNP-A *is yet to be proven to be induced at the protein level in response to pathogens, elevated protein levels have been shown as a result of abiotic stresses [15]. In addition, transcription of *AtPNP-A *is low under control conditions but strongly induced in response to biotic and abiotic stresses and the protein has been identified and isolated from the *A. thaliana *apoplast together PR-1, PR-2 and PR-5 proteins [16]. AtPNP-A has other features characteristic of PR proteins including an N-terminal signal peptide [43] that directs the molecule into the extracellular space. Further, induction of *AtPNP-A *at the transcript level appears to occur independent of *de novo *protein synthesis characteristic of genes encoding secreted proteins [28]. The evolutionary history of AtPNP-A suggests that PNPs, like the related expansins, derived from ancestral family-45 endoglucanases that have lost their hydrolytic activity and have sub-functionalized into extracellular, systemically mobile signalling molecules [9].

### Future directions

In order to determine the physiological role of *AtPNP-A *in *A. thaliana *a T-DNA insertion mutant, that is available from SALK, could be used. Phenotyping this mutant in response to SA-inducing abiotic and biotic stresses as well as during developmental senescence, will help characterise specific physiological processes in which *AtPNP-A *is involved. If such a mutant demonstrated a compromised SAR response, it would greatly strengthen the claim that *AtPNP-A *is indeed involved in the SAR response pathway. Additionally, it will be interesting to look at the expression of the correlated genes in an *AtPNP-A *mutant in response to SAR inducing conditions since this may enable us to determine a role for *AtPNP-A *in the context of a SAR response pathway.

## Conclusion

AtPNP-A is an annotated "signal" molecule that is secreted into the apoplastic space and has been implicated with a role in the control of ion and solute movements in plants (Figure 1). The expression of *AtPNP-A *is significantly correlated with that of genes involved in the SAR defence response pathway in response to various biotic and abiotic stimuli and in mutant studies. The expression of *AtPNP-A *is correlated with the *ICS-1 *gene that is involved in SA biosynthesis and *NPR1 *and *NIMIN1*, which are key positive regulators of the SAR response, and two annotated SAR TFs *TGA 3 *and *WRKY 70*. Additionally, like the PR genes, the promoter of *AtPNP-A *contains *as-1 *and W-box *cis*-elements that correspond to binding sites for the TGA 3 and WRKY TFs. Further, over expressing *WRKY 70 *lines have been shown to cause a greater than 50-fold increase in the expression of *AtPNP-A *which is consistent with this TF being a positive regulator of *AtPNP-A *transcription. The induced expression of *AtPNP-A *by SAR elicitors and its secretion into the apoplast is similar to that of PR proteins and strongly implicates a role for *AtPNP-A *in plant SAR defence responses which may involve the modification of cellular ion and water homeostasis during stress responses.

## Methods

### Identification of Correlated Genes

We downloaded (01/07/2005) *A. thaliana *gene expression levels for 1877 experiments from the NASCArrays database [81], using the bulk data download option. Perl scripts were used to calculate non-parametric correlation coefficients (Spearman's rho) between the expression of *AtPNP-A *(At2g18660) and each of the approximately 22,000 genes represented on the Affymetrix array that was used to generate this data set. We ranked genes according to the correlation coefficient and reported genes that were most positively correlated with At2g18660. The p-values were calculated using the bivariate normal distribution, with p representing the probability of observing an equal or larger positive or negative correlation by chance.

### Functional Classification and expression analysis of Correlated Genes

To characterise the correlated genes the web-based 'FatiGO+' program [22] was used to search for differential distributions of gene ontology (GO) and biological terms within this list. The search was conducted using *AtPNP-A *(At2g18660) and the 25 most positively correlated genes in Table 1 (list 1 = 26 genes). This list was compared to a reference gene list that contained the remaining genes in the entire *A. thaliana *genome (list 2 = 26147 genes). Statistical significance was determined using the Family Wise Error Rate (FWER) to calculate the adjusted p-value.

The expression profiles of *AtPNP-A *and the positively correlated genes (Table 1) were initially examined using Affymetrix public microarray data in the gene response viewer tool (GRV) in Genevestigator [68]). The analysis was performed using the ATH1: 22K array chip type and included all of the available 2507 chip sources. For better temporal and spatial response resolution we obtained normalised microarray data from the following sites: NASCArrays, Ozone-26 (reference ID); *P. infestans*-123; UV-B stress-144; Potassium starvation-105; BHT-392. TAIR (ATGenExpress): Salicylic acid-ME00364; *E.orontii*-ME00354; Salt stress-ME00328; Osmotic stress-ME00327; Cold acclimation-ME00369; Cyclohexamide-ME00361. GEO (NCBI): *E. cichoracearum*-GSE431.

In order to further reveal the relationship of *AtPNP-A *expression with that of key genes involved in the SAR response, the mutant surveyor in Genevestigator was used to compare gene expression in different types of defence related *A. thaliana *mutants. The genes investigated in this study included *AtPNP-A*, the 25 correlated genes, and *WRKY 70 *and *WRKY 46*. Normalised array data from the mutant experiments were obtained from: TAIR-ME00373 for *cpr5/npr1 *mutants; NASCarray-52 for the *nahG *mutant and array express (EBI) for the *mpk4 *mutant (E-MEXP-173). For the *WRKY 70 *over expresser, data was obtained through personal communication with Gunter Brader, Faculty of Biosciences, University of Helsinki.

### Promoter analysis

The web-based Athena [59] and POBO [58]) applications were used to analyse the promoters (-1 kb upstream of the predicted TSS) of *AtPNP- *and the 25 top correlated genes.

In POBO [58], the 1 kb promoter sequences were uploaded and the analysis was run against *A. thaliana *background (clean) searching for the TTGAC W-box core motif using the default settings (number of sequences to pick-out = 50, number of samples to generate = 1000, sequence length = 1000 bps). A two-tailed p-value was calculated in the linked online GraphPad web-site using the generated t-value and degrees of freedom to determine the statistical differences between input sequences and background.

In Athena, the analysis was performed with the visualisation tool using the 26 correlated genes with settings of 1000 bp upstream and do not cut off at adjacent genes. The statistical significance of over-represented TF binding sites is automatically calculated using a hypergeometric probability model to calculate the p-value. A Bonferroni correction was automatically used in Athena to account for multiple hypotheses testing (up to 105 different TF binding sites) and determined that the p-value threshold for significant enrichment was < 10^-4^.

## Abbreviations

NP – natriuretic peptide; PNP – plant natriuretic peptide; SAR – systemic acquired resistance; cGMP – guanosine 3',5'-cyclic monophosphate; TF – transcription factor; TSS – translation start site.

## Authors' contributions

The project was conceived by CG and VB. StM, RB, LD and ShM have extracted and interpreted the data. The manuscript was written by StM and CG. All authors read and approved the final manuscript.

## Supplementary Material

Additional file 1 GO analysis of *AtPNP-A *expression correlated genes. List of significantly enriched GO terms associated with *AtPNP-A *(At2g18660) expression correlated genes in FatiGO+. Click here for file

Additional file 2 Extended list of genes expression correlated with *AtPNP-A*. An extended list containing all genes that are expression correlated with *AtPNP-A *(At2g18660) and a list of all annotated SAR genes in *Arabidopsis thaliana*. Click here for file

Additional file 3 Promoter analysis. AtPNP-A (At2g18660) and the expression correlated genes were analysed in POBO and Athena for the presence of W-boxes. Click here for file
