# Supplementary material for: Co-expression and promoter content analyses assign a role in biotic and abiotic stress responses to plant natriuretic peptides
Source: BMC Plant Biol. 2008 Feb 29;8:24. doi: 10.1186/1471-2229-8-24 (PMC2268938; doi:10.1186/1471-2229-8-24)
Supplement: Additional file 1 — GO analysis of AtPNP-A expression correlated genes. List of significantly enriched GO terms associated with AtPNP-A (At2g18660) expression correlated genes in FatiGO+. [file 1471-2229-8-24-S1.pdf]

FatiGO+ analysis

Gene Ontology :

Summary input data: Biological process terms: 1940

Genes in list 1 used in analysis: 26 No. genes with annotation in list 1: 15

Genes in list 2 used in analysis: 26106 No. genes with annotation in list 2: 11192

		List 1		List 2		Significance
GO term	Level	No. genes	%	No. genes	%	Adj. p value
Resp. to biotic stim.	3	6	40	294	2.6	$1.57e^{-03}$
Immune system process	3	4	26.6	108	0.9	$5.52e^{-03}$
Resp. to other organism	4	6	42.8	289	2.7	$1.57e^{-03}$
Immune response	4	4	28.5	108	1.0	$5.52e^{-03}$
Innate immune response	5	4	36.3	104	1.0	$2.78e^{-03}$
Innate immune resp.	6	4	44.4	102	1.2	$2.23e^{-03}$
Def. resp, incompat int.	7	4	50	73	1.1	$1.57e^{-03}$
SAR	8	4	100	17	0.4	$3.84e^{-06}$

No. genes = number of genes in the specified list associated with the GO term at the indicated level.
% = percentage of genes in a list with annotation at the indicated level which are associated with a particular GO term. For example: in list 1, 4 genes have annotation at level 8 and these are all associated with SAR thus the percentage is 100.

Swiss-Prot keyword search

SwissProt keywords: 387

Number of genes with annotation in list 1: 7

Number of genes with annotation in list 2: 3766

	List 1		List 2		Significance
Swiss-Prot keyword	No. genes	%	No. genes	%	Adj. p value
PR-protein	3	42.86	15	0.4	$2.23e^{-03}$
Signal	6	85.71	550	14.6	$2.57e^{-02}$
Apoplast	3	42.86	53	1.41	$3.96e^{-02}$

% = percentage of annotated genes associated with the Swiss-prot keywords.
Adjusted p-value = Family Wise Error Rate (FWER).
