# Supplementary material for: Co-expression and promoter content analyses assign a role in biotic and abiotic stress responses to plant natriuretic peptides
Source: BMC Plant Biol. 2008 Feb 29;8:24. doi: 10.1186/1471-2229-8-24 (PMC2268938; doi:10.1186/1471-2229-8-24)
Supplement: Additional file 3 — Promoter analysis. AtPNP-A (At2g18660) and the expression correlated genes were analysed in POBO and Athena for the presence of W-boxes. [file 1471-2229-8-24-S3.doc]

**PROMOTER ANALYSIS**

**POBO analysis results summary**

# SETTINGS

Search pattern = TTGAC

Background (BG) model: *Arabidopsis_thaliana*_clean

number of sequences to pick-out = 50 (default)

number of samples (pseudoclusters) to generate = 1000 (default)

sequence length = 1000 bps

Cluster 1 input = promoter sequences (-1kb relative to TSS) for 26 expression correlated genes

**RESULTS**


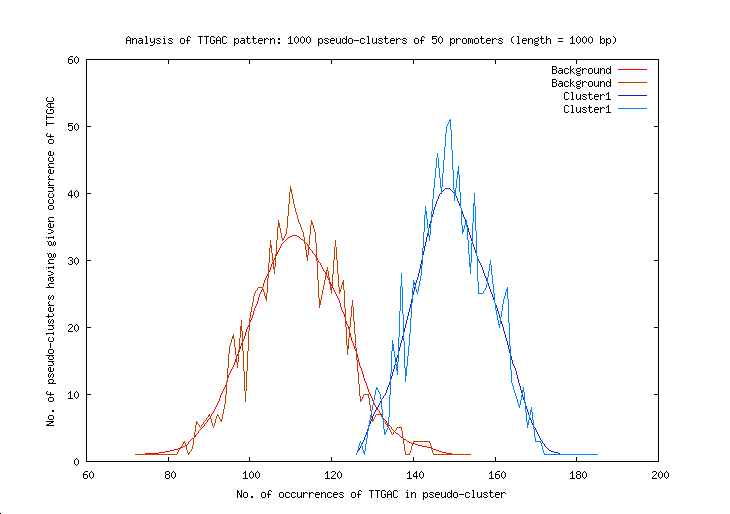


FIGURE 1: Illustrates the frequency of the occurrence of the search pattern (TTGAC) in

the artificial clusters generated from the background and input sequences.

| Data set | Number of prom. in a dataset | Number of promoters containing the pattern | Total number of patterns in dataset | Promoter mean |
| --- | --- | --- | --- | --- |
| BG  model | 31353 | 27523 (87.8%) | 70245 | 2.24 |
| Cluster1 | 26 | 25 (96.2%) | 78 | 2.99 |

**STATISTICS**

Independent T-test between Cluster 1 and background

t-value = 81.60, Degrees of Freedom =1998.

The two-tailed P value (calculated using the linked online GraphPad web-site; http://www.graphpad.com/quickcalcs/DistMenu.cfm) was determined to be less than 0.0001 indicating the difference between samples is extremely statistically significant.

**ATHENA promoter analysis**

| **Search sequence** | **No. promoters searched** | **No. promoters with site** | **Total No. sites** | **Av. copies /promoter** | **P-value** |
| --- | --- | --- | --- | --- | --- |
| **W-box TTGAC(A/T)** | **26** | **22 (85%)** | **54** | **2.08** | **0.0037** |

The W-box sequence **TTGAC(A/T)** is not considered enriched in these genes since the p-value is not below the stringent threshold P-value of <10-4 to qualify as enriched.
